# Development of the Chinese Version of Medication Adherence Reasons Scale (ChMAR-Scale)

**DOI:** 10.3390/ijerph17155578

**Published:** 2020-08-02

**Authors:** Pin-Fang Chen, Elizabeth H. Chang, Elizabeth J. Unni, Man Hung

**Affiliations:** 1 Department of Clinical Pharmacy, School of Pharmacy, College of Pharmacy, Taipei Medical University, Taipei 110301, Taiwan; fannychen219@gmail.com; 2 Department of Pharmacy, Wan Fang Hospital, Taipei Medical University, Taipei 116081, Taiwan; 3 Research Center for Pharmacoeconomics, College of Pharmacy, Taipei Medical University, Taipei 110301, Taiwan; 4 Department of Social, Behavioral, and Administrative Sciences, Touro College of Pharmacy, New York, NY 10027, USA; elizabeth.unni@touro.edu; 5 College of Dental Medicine, Roseman University of Health Sciences, South Jordan, UT 84095, USA; mhung@roseman.edu

**Keywords:** medication adherence, surveys and questionnaires, validation study, hypertension

## Abstract

Medication non-adherence is a concern in chronic disease management. Currently, there is no scale that characterizes sufficient non-adherent reasons for practical use in the Chinese population. This study developed and validated the Chinese version of the Medication Adherence Reasons Scale (ChMAR-Scale) and described non-adherence reasons in adult patients taking blood pressure medicine in Taiwan. A forward–backward procedure was used to translate the original MAR-Scale, and new items pertinent to cultural differences were added. Patients aged above 20 years old who were taking blood pressure medicine were recruited from a regional hospital and eight community pharmacies in the Taipei metropolitan area. Data analyses were conducted with IBM SPSS 19 (Armonk, NY, USA). Exploratory factor analysis revealed six domains, including belief, self-perception, forgetfulness, management, availability, and miscellaneous issues, with Cronbach’s alphas ranging from 0.649 to 0.852, item-total correlations ranging from 0.362 to 0.719, and factor loadings ranging from 0.365 to 0.775. Criterion-related validity with the visual analog scale and two global items were 0.525, 0.436, and 0.502. Forgetfulness, belief issues, and self-perception issues were the most common non-adherence reasons. In conclusion, the ChMAR-Scale showed good psychometric properties and identified more reasons for medication non-adherence than other existing scales. Healthcare providers should be vigilant of these problems while consulting patients.

## 1. Introduction

Medication adherence is important in patients with chronic diseases. These are defined as “conditions that last one year or more and require ongoing medical attention or limit activities of daily living or both” and broadly include diseases such as high blood pressure, stroke, cancer, and chronic lung disease [1]. Chronic diseases often require patients to take medications throughout their life. Without proper adherence to prescribed medications, patients are prone to progression of disease, death, and increased healthcare use and costs [2], and research showed that, after the first six months of medication therapy for chronic diseases, the rates of medication adherence drop substantially, by as much as 50% [3,4]. Past research showed that the prevalence of medication non-adherence in East Asia ranges from 19.8 to 51.0% across chronic diseases of epilepsy, stroke, diabetes, and high blood pressure [5,6,7,8]. The reasons for non-adherence mostly corresponded to the five dimensions of adherence defined by the World Health Organization [9], including social and economic factors, healthcare team and system-related factors, condition-related factors, therapy-related factors, and patient-related factors. In particular, patient-related factors are especially complex due to difficulties in measurement and limited effective interventions.

Several instruments for measuring medication adherence have been developed [10,11,12,13,14,15]. These instruments mostly measure barriers to adherence, adherence to recommendations, and adherence behaviors in patients with chronic diseases via self-administered questionnaires. However, limitations of existing scales have been identified [16,17], such as limited reasons for non-adherence captured leading to difficulty for targeted intervention. If the scale is too brief to report the extent of non-adherence and reveal the reasons for non-adherence, the non-adherence rate may be underestimated and the issues contributing to non-adherence may remain unresolved.

To improve upon the limitations of earlier scales, Unni and colleagues [17,18] developed the Medication Adherence Reason Scale (MAR-Scale), which contains a comprehensive list of reasons for non-adherence that can be used to inform issues including belief, self-perception, forgetfulness, management, and availability. It has been tested in patients with high cholesterol and asthma in the U.S., with good psychometric properties. An updated MAR-Scale has been further validated in 17 different chronic conditions with acceptable reliability, which shows its utility to capture reasons for medication non-adherence across disease states [19].

Currently, there are limited scales that aim to provide reasons for non-adherence to blood pressure medications in the Chinese population. Although several Chinese versions were used in measuring adherence to medication in patients with epilepsy, myocardial infarction, and high blood pressure, these are limited in recognizing the potential reasons for non-adherent behaviors in patients [5,20,21,22]. Therefore, to develop a new adherence scale that is adapted to cultural and healthcare system differences is in demand. The objectives of this study are twofold: (1) translate the MAR-Scale into Chinese and examine its psychometric properties and cultural adaptation among patients with high blood pressure in Taiwan; (2) characterize medication non-adherence issues among these patients using the Chinese version of the Medication Adherence Reasons Scale (ChMAR-Scale).

## 2. Materials and Methods

**Figure 1:**
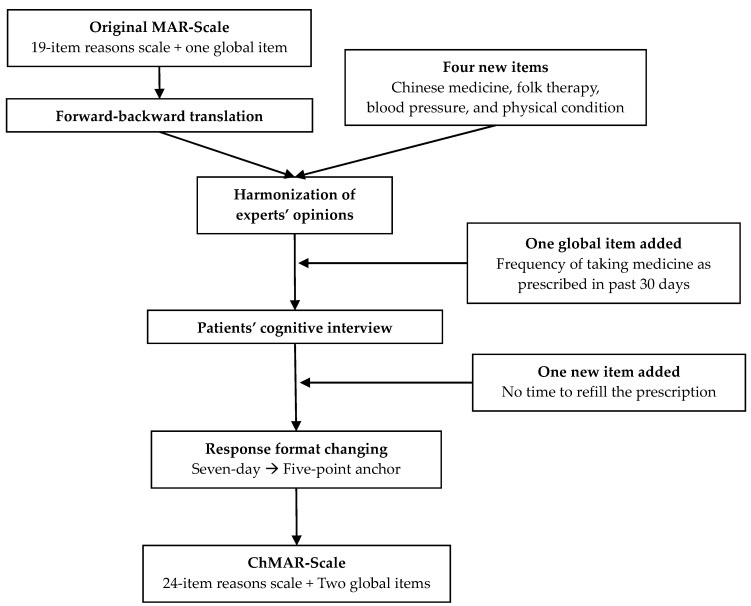
Diagram for the development of the Chinese version of Medication Adherence Reasons Scale (ChMAR-Scale).

The original MAR-Scale was translated into Chinese (Figure 1) following the “Principles of Good Practice for the Translation and Cultural Adaptation Process for Patient-Reported Outcomes (PRO) Measures” [23]. Forward translation of the English version of the MAR-Scale to Chinese was performed by two independent translators who were native speakers of Chinese and proficient in English. After reconciliation, a bilingual pharmacist with a doctor of pharmacy degree back-translated the forward translation into English. The researchers then compared the forward and backward translations and the original text and conducted another round of reconciliation. To ensure content validity, five subject experts (three professors with administrative pharmacy specialty and two hospital pharmacists) were asked to review the translation appropriateness of the “pre-expert review” version and assess the cultural adaptation of each item. The experts recommended changing the response format from a seven-day anchor to a five-point anchor. Another reconciliation was conducted after obtaining the experts’ opinions. The draft of the questionnaire was then distributed to 10 adults with high blood pressure for cognitive interviews based on convenience sampling. A “think aloud process” was used to assess the level of their comprehensibility of each item of the ChMAR-Scale. The patients were asked to complete both the seven-day anchor and five-point anchor scales to determine which of the response formats they would be more likely to complete. After modifying the questionnaire and proofreading, the final version was completed.

At least six revisions of the questionnaire were produced after the initial translation. Firstly, the response format was changed to a five-point scale due to the experts’ opinions and evidence that respondents had difficulty filling out the original seven-day response format. Secondly, the scale focused on medication use over the past month rather than week, as several items were not applicable when asking only about the past week, such as the frequency of medication pick-up, which is typically once monthly. Thirdly, new items based on past research regarding cultural differences, such as “Chinese Medicine or herbal use, folk therapy, change dosage according to blood pressure (BP) or physical condition”, were added into the ChMAR-Scale. “No time to refill the prescription in the pharmacy” was also added to the scale based on cognitive interviews. Fourthly, one additional five-point global item asking, “Over the last 30 days, how often were you able to take your blood pressure medicine exactly as prescribed?” was added. Finally, during the cognitive interviews, several patients filled in the items asking “how often they had this kind of thought” but not “how often they were unable to take the medicine because of these reasons” after cognitive interview. To avoid this confusion, we added a sentence, “The frequency you missed taking medicine as prescribed due to various reasons of the following items”, above every item. The final ChMAR-Scale includes 24 items of non-adherent reasons and two global items of non-adherence.

A cross-sectional survey design was used to validate the data. The survey questionnaire had 45 items, which included medication non-adherence (Visual Analog Scale (VAS), ChMAR-Scale); patient demographics (sex, age, education level, monthly salary, smoking history); disease characteristics (comorbidity and most recent BP measurement); treatment characteristics (treatment duration, regimen, dosing, and Chinese medicine, herbal, and folk therapy use). Following previous MAR-Scale studies, participants were categorized as non-adherent if they did not answer “never” on one or more items. BP was considered controlled if (1) systolic/diastolic BP < 140/90 mmHg in patients with diabetes or chronic kidney disease; (2) systolic/diastolic BP < 140/90 mmHg in patients without diabetes or chronic kidney disease and <60 years old; (3) systolic/diastolic BP < 150/90 mmHg in patients without diabetes or chronic kidney disease and ≥60 years old [24].

The Visual Analog Scale (VAS) developed by Nau et al. was used to validate the new scale [25]. The VAS has a single item asking, “What percentage of time over the past 30 days did you take your prescribed blood pressure medicine?” The individuals were guided to mark an “X” on a horizontal line which was anchored by 0% and 100%. The VAS was translated into Chinese by the researchers. Thirteen individuals with pharmacy backgrounds and four lay-persons were asked to read the translated VAS. The Chinese VAS was finalized based on the feedback from these experts.

A cross-sectional design was used, and the study was conducted between February and May 2016. The subjects were recruited in a regional hospital and eight community pharmacies in the Taipei metropolitan area to maintain diversity among patients. The inclusion criteria of the study were (1) 20 years or older and diagnosed with high blood pressure by a physician and (2) people who had taken blood pressure medicine. The exclusion criterion was an inability to communicate in Chinese.

The recruitment was conducted based on convenience sampling by P.F.C. in the hospital, while the recruitment in the community pharmacies was conducted by the community pharmacists. The community pharmacists were individually trained based on a page of standardized instructions about the criteria for recruiting patients, and they were given one reminder about recruiting after a month. Patients were recruited if they were picking up prescriptions for blood pressure medicine and showed an interest in participation after a brief explanation of the study. The recruiters assisted patients in answering the questions regarding which blood pressure medicine they were using. In addition, patients who could not read Chinese were assisted by the recruiters by reading each item for them. Two screening questions were asked at the beginning of the questionnaire to confirm that patients met the two inclusion criteria described above. A gift equivalent to $1 USD was provided as an incentive to each patient completing the questionnaire. The data were transcribed and checked by different investigators to ensure accuracy.

Descriptive analysis was completed to examine the demographic data and responses in the ChMAR-Scale. Item analysis, factor analysis, cross-validation, and Cronbach’s alpha were conducted to assess construct validity and reliability [26,27]. Principle axis factoring, varimax rotation, and the Kaiser criterion were used to examine the underlying factors in the exploratory factor analysis. Cramer’s V and the kappa coefficient were used to examine the criterion-related validity between the new scale and the Chinese VAS, Global Item 1 (taking medicine for the past seven days), and Global Item 2 (adherence to the prescription over the past 30 days) [28]. Listwise deletion was used for missing data. For validation purposes, adherence measures were dichotomized, and non-adherence was defined for each item as the following: (1) participants who did not answer “never” on at least one item in the ChMAR-Scale, (2) those who did not answer “100%” in the VAS, (3) participants who did not state that they took seven days of blood pressure medicine as prescribed over the past week in Global Item 1, and (4) participants who did not answer that they had adhered consistently over the past 30 days in Global Item 2.

The research protocol was approved by the Taipei Medical University Joint Institutional Review Board (N201512008) with a waiver of informed consent.

## 3. Results

A total of 621 patients were recruited and 574 questionnaires were returned, with an effective sample size of 538 (response rate 86.6%). A total of 64% of the questionnaires were collected in the hospital and 36% were collected in community pharmacies. A total of 36 questionnaires were excluded because respondents did not meet the inclusion criteria for the study for the following reasons: (1) patients not diagnosed with high blood pressure or (2) patients not taking blood pressure medicine. Over half of our respondents were male (55.4%) and less than 65 years old (53.2%) (Table 1). Participants with less than high school education accounted for a significant proportion (40.3%). Over half of participants (50.3%) had taken blood pressure medicine for more than six years; most of them took the blood pressure medicine once a day (72.8%) and one or less pill (59.6%). Based on the ChMAR-Scale, 61.7% of the respondents were non-adherent with their blood pressure medicine. Overall, 67.5% of the participants had BP under control. In the adherence group, 74.9% had the BP under control, which was significantly higher than the non-adherence group (Table 2).

### 3.1. Criterion-Related Validity

In the comparative analysis of the ChMAR-Scale and the VAS, the correlation of the two scales was 0.525 and was significant at the level of 0.001. The performance of the ChMAR-Scale in comparison to the VAS and two global items is listed in Table 3. There were 142 patients identified as non-adherent by the ChMAR-Scale, but they were identified as adherents by the VAS. Comparatively, there were 10 participants recognized as adherents by the ChMAR-Scale, but they were identified as non-adherents by the VAS. In the comparison of the ChMAR-Scale and the global items, the correlations were 0.436 with Global Item 1 and 0.502 with Global Item 2. Both were significant at the level of 0.001.

### 3.2. Construct Validity

The Kaiser criterion suggested six factors to be retained in the ChMAR-Scale, and the total variance explained by the factors was 56%. The first factor reflected the concern or belief about social or personal issues that hindered adherence and was named “belief issues”. The second factor was named “self-perception issues” and reflected the idea of controlling their condition based on their physical condition and how they felt. The third extracted factor dealt with non-adherence due to several “forgetfulness” issues. The fourth factor reflected the issue of “managing issues”. “Availability issues” was the fifth extracted factor, reflecting the issue of difficulty in obtaining prescribed medicine in the pharmacy. The last three items about Chinese medicine, folk therapy, and cost issue were named “miscellaneous”, constituting the sixth factor. The factor loading for each item was above acceptable levels and ranged from 0.365 to 0.775 (Table 4).

### 3.3. Reliability

The six domains had acceptable Cronbach’s alpha values ranging from 0.649 to 0.852, all above the acceptable value of 0.6. The item-total correlation ranged from 0.362 to 0.719 and demonstrated good reliability. Item analysis suggested dropping items Q11, Q21, and Q24; however, the three items were all retained because of conceptual importance.

### 3.4. Quantifying Non-Adherence

The ChMAR-Scale was able to identify more non-adherence to medication-taking behavior than the VAS: the ChMAR-Scale identified 61.6% of the respondents as non-adherent while the VAS identified 36.9% as non-adherents. Global Items 1 and 2 only identified 24.1% and 36.4% of participants as non-adherents, respectively.

In the responses to the VAS, the mean adherence score was 89.3%. Most respondents (63.1%) were being adherent “100% of the time” over the last 30 days in the VAS. In Global Item 2, 63.6% of participants indicated that they “always” were able to take the blood pressure medicine exactly as prescribed over the last 30 days. The responses of “always” to Global Item 2 and “100%” in the VAS were consistent. When asked about adherence over the last seven days, most (75.9%) reported that they were able to take their blood pressure medicine every day. The average number of days that participants were able to follow the physicians’ prescription was 3.46 (SD: 0.902), 51% of the time. Patients reported better adherence in a shorter interval (past seven days) than in a longer interval (past 30 days).

Forgetfulness issues were the most common non-adherence reasons among the participants. A total of 36% of the respondents simply missed a dose (Q12), 30.1% had problems forgetting things in daily life (Q13), and 27.5% forgot to take blood pressure medicine due to a busy life (Q14). The next most common reason was belief issues. Many of the participants were non-adherent because of worrying about the side effects (Q1: 26.4%) or the long-term effects (Q2: 27.7%) of the blood pressure medicine. A total of 27% of the respondents were non-adherent due to personal reasons. Additionally, many of the participants stopped taking their blood pressure medicine to see if they still needed it (Q3:24%). Around 22% reported changing the usage of their blood pressure medicine based on their physical condition (Q9: 22.7%) and BP (Q10: 22.1%). The least common reasons for non-adherence were using a non-traditional therapy (Q22: 3.2%; Q23: 5%), the pharmacy having no supply of medicine (Q21: 5%), and cost issues (Q24: 4.5%).

## 4. Discussion

The ChMAR-Scale was translated from the MAR-Scale and modified based on cultural and healthcare system differences, and its criterion-related validity, construct validity, and reliability each showed acceptable to good psychometric properties. Moreover, the ChMAR-Scale identified more patients with non-adherence than other global items in this study, signifying the value of the new scale in identifying patients needing further attention for medication adherence issues.

Compared to previous validated adherence scales in Chinese, the 24-item ChMAR-Scale provides a comprehensive framework addressing more belief, perception, and management as well as availability issues in medication-taking behaviors. As such, the ChMAR-Scale can serve as a screening tool prior to appointments with healthcare providers, which can not only prepare the patient to ask more questions about their medications but also allow the healthcare provider to examine whether there are areas for improvement in terms of the complexity of treatment and social support needed. This is especially important in the Chinese community because of the large patient volume typically seen in hospitals, leading to limited time per visit as well as patients’ tendency to be conservative and avoid conflicts in communications based on cultural roots [29].

In addition to serving as a screening tool for healthcare providers, the ChMAR-Scale has the potential to be used as a follow-up and research tool for evaluating the long-term effectiveness of counseling interventions aimed at improving medication adherence. Given that many personal and healthcare system barriers addressed in the ChMAR-Scale are not easy to address in a single visit, the tool can be helpful in developing a long-term plan for improving medication adherence. Future studies could not only examine the sensitivity of the ChMAR-Scale in capturing intervention effectiveness but also examine how healthcare providers can better prioritize how to address different reasons for non-adherence.

The ChMAR-Scale showed psychometric properties similar to the original MAR-Scale, with good factor loadings, item-total correlations, and acceptable Cronbach’s alphas. A major difference is that the ChMAR-Scale identified six domains whereas the original MAR-Scale only had four domains. While belief issues, forgetfulness, and managing issues in the ChMAR-Scale directly correspond to existing domains in the original scale, the ChMAR-Scale highlighted factors regarding self-perception issues, availability, and miscellaneous issues. This finding is reasonable due to two reasons: firstly, there were purposive efforts to culturally adapt the instrument based on differences in healthcare systems and cultural needs in this study, and the findings support the need for the additional items. Secondly, the number of factors contributing to non-adherence may be different across disease states. For example, in this study, blood pressure is a visible outcome for patients to self-manage at home; however, in the validation of the original MAR-Scale, patients used cholesterol lowering and asthma medication as preventive treatments, which are more intangible. Therefore, self-perceptions might have stronger influences on medication adherence in patients with high blood pressure.

Based on the characteristics of non-adherence behaviors in this sample, the most common reasons were forgetfulness, concerns about side effects or long-term effects, and self-adjustment of medications according to BP, physical condition, or to check whether it was still needed. In the US, forgetfulness issues, patients’ concerns about side effects or long-term effects, and cost concerns were also common non-adherence reasons [30]. One explanation of the differences in cost concern, which is a less important reason for non-adherence in Taiwan, is likely due to most blood pressure medications being covered by National Health Insurance.

Except for the cost issue, few people stop their blood pressure medications to switch to alternative therapy. The reason may be the low responses of non-adherence due to our sampling methods. People who visit the hospital or pharmacy may be less likely to seek non-traditional therapy. Another reason is that people may view non-traditional therapy as interfering with blood pressure medications.

There were limitations in this study. Firstly, data were only collected at one hospital and eight pharmacies in the Taipei metropolitan area, thus limiting the generalizability. Secondly, recall bias, which means recalling information more or less compared to actual situations, might occur as many respondents were not familiar with their medications. Thirdly, social desirability bias, which means the respondent reports favorable answers to researchers, might have influenced the self-reported results to be different from reality. Fourthly, blood pressure values of the most recent measurement were based on self-report and underestimation of blood pressure control is likely. Nevertheless, participants were encouraged to use hospital or pharmacy devices whenever possible. Finally, while we showed reliability in terms of internal consistency measures; we were unable to conduct test-retest reliability due to practical issues in recruitment.

## 5. Conclusions

The new ChMAR-Scale identified more comprehensive reasons for non-adherence and showed good validity and acceptable to good reliability. It can be incorporated into practice settings for screening and follow-up purposes to facilitate communications between healthcare providers and patients. This study also identified forgetfulness, belief issues, and self-perception issues as common reasons for non-adherence and can be informative for healthcare providers while consulting patients. Future studies could further examine the scale in different disease conditions among variable populations and settings and test the sensitivity of the scale to interventions by healthcare providers.

## Figures and Tables

**Table 1:** Participants’ characteristics.

Characteristics	N	%
Male	292	55.4
Age < 65	284	53.2
Education less than high school	215	40.3
**Duration of Taking Blood Pressure Medicine**		
<1 year	41	7.6
1~2 year(s)	78	14.5
3~5 years	143	26.6
6~9 years	96	17.8
≥10 years	180	33.5
**Frequency of Taking Blood Pressure Medicine**		
1 time/day	385	72.8
2 times/day	109	20.6
3 times/day	23	4.3
4 times/day	3	0.6
Don’t know	9	1.7
**Blood Pressure Medicine Pills Per Time**		
0.5~1 #	311	59.6
1.5~2 #	133	25.5
2.5~3 #	42	8.0
≥3.5 #	19	3.6
Not sure	17	3.3

**Table 2:** Blood pressure (BP) control rate vs. adherence based on the ChMAR-Scale.

	ChMAR-Scale ^1^						*p*
	Adherence		Non-Adherence		Total		
	N	%	N	%	N	%	
Not controlled ^2^	49	25.1	108	37.5	157	32.5	<0.005
Controlled	146	74.9	180	62.5	326	67.5	
Total	195	100	288	100	483	100	

^1^ Non-adherence based on ChMAR-Scale was defined as not answering “never” on any item. ^2^ BP was considered not controlled if (1) systolic/diastolic BP ≥ 140/90 mmHg in patients with diabetes or chronic kidney disease; (2) systolic/diastolic BP ≥ 140/90 mmHg in patients without diabetes or chronic kidney disease and <60 years old; (3) systolic/diastolic BP ≥ 150/90 mmHg in patients without diabetes or chronic kidney disease and ≥60 years old.

**Table 3:** Criterion-related validity of the ChMAR-Scale.

Variable	ChMAR-Scale ^1^					
	Adherent		Non-Adherent		Total	
	N	%	N	%	N	%
**Visual Analog Scale (VAS): What percentage of time over the past 30 days did you take your prescribed blood pressure medicine?**						
Adherent	196	95.1	142	43.0	338	63.1
Non-adherent ^2^	10	4.9	188	57.0	198	36.9
Total	206	100.0	330	100.0	536	100.0
Cramer’s V: 0.525 **						
Kappa score: 0.465 **						
**Global Item 1: Over the last seven days, how many days were you able to take your blood pressure medicine exactly as prescribed?**						
Adherent	205	99.5	202	61.2	407	75.9
Non-adherent ^3^	1	0.5	128	38.8	129	24.1
Total	206	100.0	330	100.0	536	100.0
Cramer’s V: 0.436 **						
Kappa score: 0.324 **						
**Global Item 2: Over the last 30 days, how often were you able to take your blood pressure medicine exactly as prescribed?**						
Adherent	194	94.2	147	44.5	341	63.6
Non-adherent ^4^	12	5.8	183	55.5	195	36.4
Total	206	100.0	330	100.0	536	100.0
Cramer’s V: 0.502 **						
Kappa score: 0.442 **						

** *p* < 0.001. ^1^ Non-adherent in ChMAR-Scale: did not answer “never” on any item. ^2^ Non-adherent in VAS: VAS < 100%. ^3^ Non-adherent in Global Item 1: participants who did not answer taking seven days of blood pressure medicine as prescribed over the past week. ^4^ Non-adherent in Global Item 2: participants who did not answer that they had adhered all of the time over the past 30 days.

**Table 4:** Psychometric properties of the ChMAR-Scale ^1^.

Over the Last 30 Days, When You Were Not Able to Take Your Blood Pressure Medicine as Prescribed, How Often Did It Happen for Each of the Following Reasons?	Responses (%; N = 538 Unless Noted)					Non-Adherence (%)	Factor Loading N = 533	Item-Total Correlation N = 533	Cronbach’s Alpha
	Never	A Little	Some	Most	All of the Time				
**Factor 1: Belief issues**									0.852
Q1. I am concerned about possible side effects from this medicine	73.6	10.2	9.9	4.8	1.5	26.4	0.781	0.673	
^2^ Q2. I am concerned about long term effects from this medicine	72.3	10.4	10.6	5.0	1.7	27.7	0.758	0.660	
Q3. I sometimes skip this medicine to see if it is still needed	76.0	11.0	9.1	2.6	1.3	24.0	0.530	0.719	
Q4. I had side effects from this medicine	79.9	15.1	3.9	0.7	0.4	20.1	0.480	0.637	
^2^ Q5. I was not comfortable taking it for social reasons (e.g., I was with friends)	87.7	9.3	2.4	0.4	0.2	12.3	0.451	0.497	
^3^ Q6. I don’t think that this medicine is working for me	86.4	8.6	3.7	0.2	1.1	13.6	0.429	0.559	
Q7. I was not comfortable taking it for personal reasons (e.g., I was travelling)	72.9	16.4	9.5	1.3	0	27.1	0.421	0.487	
Q8. I do not consider taking this medicine as a high priority in my daily routine	82.5	10.8	5.2	0.6	0.9	17.5	0.406	0.450	
**Factor 2: Self- perception issues**									0.83
^1^ Q9. I adjusted medicine according to my physical condition	77.3	11.9	5.6	3.5	1.7	22.7	0.882	0.633	
^1^ Q10. I adjusted medicine according to my blood pressure	77.9	9.9	7.2	3.2	1.9	22.1	0.816	0.598	
Q11. I don’t think that I need this medicine anymore	82.5	8.4	5.6	2.0	1.5	17.5	0.435	0.535	
**Factor 3: Forgetfulness**									0.842
^2^ Q12. I would have taken it but simply missed it	63.7	20.7	12.1	2.6	0.9	36.3	0.886	0.516	
Q13. I would have taken it but have problems forgetting things in my daily life	69.9	18.2	8.7	2.2	0.9	30.1	0.680	0.590	
Q14. I would have taken it but missed it because of busy schedule	72.5	16.5	8.7	1.7	0.6	27.5	0.655	0.581	
**Factor 4: Managing issues**									0.8
Q15. I have trouble managing all the medicines I have to take	90.0	6.3	2.8	0.9	0	10.0	0.665	0.569	
Q16. I am not sure how to take this medicine	91.3	7.2	1.1	0.2	0.2	8.7	0.592	0.516	
Q17. I had difficulty opening the container	90.7	6.5	2.0	0.7	0	9.3	0.543	0.560	
Q18. I had difficulty swallowing this medicine	89.6	8.6	1.7	0.2	0	10.4	0.501	0.492	
**Factor 5: A vailability issues**									0.76
Q19. I didn’t have the medicine because I didn’t have a ride to the pharmacy	93.7	4.5	1.3	0.4	0.2	6.3	0.889	0.405	
^1,2^ Q20. I didn’t have the medicine because I didn’t have time to go to the pharmacy	91.2	6.3	1.9	0.4	0.2	8.8	0.701	0.493	
Q21. I didn’t have the medicine because the pharmacy was out of this medicine	95.0	4.1	0.4	0.6	0	5.0	0.418	0.401	
**Factor 6: Miscellaneous**									0.649
^1^ Q22. I was also taking folk therapy so I adjusted the dosage of my blood pressure medicine	96.8	1.9	1.1	0.2	0	3.2	0.807	0.369	
^1^ Q23. I was also taking Chinese Medicine so I adjusted the dosage of my blood pressure medicine	95.0	3.0	2.0	0	0	5.0	0.515	0.362	
Q24. I did not have money to pay for this medicine	95.5	3.7	0.4	0.4	0	4.5	0.365	0.377	

^1^ Additional items added into the ChMAR-Scale that were not in the original MAR-Scale. ^2^ Items with responses of N = 537. ^3^ Items with responses of N = 536.
